# Supplementary material for: Quantum steering of Gaussian states via non-Gaussian measurements
Source: Sci Rep. 2016 Jul 14;6:29729. doi: 10.1038/srep29729 (PMC4944338; doi:10.1038/srep29729)
Supplement: Supplementary Information [file srep29729-s1.pdf]

Quantum steering of Gaussian states via non-Gaussian measurements

Se-Wan Ji,[1] Jaehak Lee,[1] Jiyong Park,[1] and Hyunchul Nha[1]

[1] *Department of Physics, Texas A&M University at Qatar University, PO Box 23784, Doha, Qatar*

SUPPLEMENTARY INFORMATION

S1. Proofs

(i) Proof of Lemma: First, a direct calculation readily gives $\sum_j \left(A_j^{(n)}\right)^2 = n\mathbb{1}_n$. On the other hand, the sum of squares of expectation values is given by

$$\begin{aligned}\sum_j \langle A_j^{(n)}\rangle^2 &= \sum_{k=0}^{n-1}\langle|k\rangle\langle k|\rangle^2 \\ &+ \tfrac{1}{2}\sum_{k<l}\left(\langle|k\rangle\langle l|\rangle^2 + \langle|l\rangle\langle k|\rangle^2 + 2|\langle|k\rangle\langle l|\rangle|^2\right) \\ &- \tfrac{1}{2}\sum_{k<l}\left(\langle|k\rangle\langle l|\rangle^2 + \langle|l\rangle\langle k|\rangle^2 - 2|\langle|k\rangle\langle l|\rangle|^2\right) \\ &= \sum_{k=0}^{n-1}\langle|k\rangle\langle k|\rangle^2 + 2\sum_{k<l}|\langle|k\rangle\langle l|\rangle|^2 \\ &\le \sum_{k=0}^{n-1}\langle|k\rangle\langle k|\rangle^2 + 2\sum_{k<l}\langle|k\rangle\langle k|\rangle\langle|l\rangle\langle l|\rangle \\ &= \langle\sum_{k=0}^{n-1}|k\rangle\langle k|\rangle^2 \le \langle\mathbb{1}_n\rangle,\end{aligned} \tag{S1}$$

where we use the Cauchy inequality to obtain the inequality in the fifth line. The positivity of the variance and $\mathbb{1}_n^2 = \mathbb{1}_n$ yield the last inequality, which in turn gives the uncertainty relation in equation (8) of main text. The equality holds when a given state is a pure state within the Fock space spanned by $\{|0\rangle, ..., |n-1\rangle\}$.

(ii) Proof of Theorem: As shown in [1], if the set of observables satisfies uncertainty relations in a sum or product form as Eq. (8) of main text, the correlation of a bipartite quantum state described by the LHS models must satisfy a non-steerablity inequality in a form

$$\sum_j \delta_{inf}^2\left(A_j^{(n)}\right) \ge (n-1)\langle\mathbb{1}_n^A\rangle, \tag{S2}$$

where $\delta_{inf}^2\left(A_j^{(n)}\right)$ is an inferred variance [2] defined by

$$\delta_{inf}^2\left(A_j^{(n)}\right) = \langle\left[A_j^{(n)} - A_{j,est}^{(n)}\left(B_j^{(n')}\right)\right]^2\rangle. \tag{S3}$$

$A_{j,est}^{(n)}\left(B_j^{(n')}\right)$ is an estimate based on Bob's outcome $B_j^{(n')}$ [1, 2]. With a choice of linear estimate $A_{j,est}^{(n)}\left(B_j^{(n')}\right) = -g_j B_j^{(n')} + \langle A_j^{(n)} + g_j B_j^{(n')}\rangle$ (g_j is an arbitrary real number) [1, 3] and setting $g = g_1 = g_2 = ... = g_n$, we obtain the non-steerability criterion in equation (9) of main text.

(iii) Proof of Proposition: Similar to the analysis in [4], let us first choose g to make the left-hand side of equation (9) of main text as small as possible, i.e.

$$g = -\frac{\sum_j\langle A_j^{(n)}\otimes B_j^{(n')}\rangle - \langle A_j^{(n)}\rangle\langle B_j^{(n')}\rangle}{\sum_j \delta^2\left(B_j^{(n')}\right)}. \tag{S4}$$

Plugging it to the inequality (9) of main text, we obtain the nonsteerability condition as

$$\begin{aligned}&|\sum_j\langle A_j^{(n)}\otimes B_j^{(n')}\rangle - \langle A_j^{(n)}\rangle\langle B_j^{(n')}\rangle| \\ &\le \sqrt{\left(\langle\mathbb{1}_n^A\rangle - \sum_j\langle A_j^{(n)}\rangle^2\right)\left(n'\langle\mathbb{1}_{n'}^B\rangle - \sum_j\langle B_j^{(n')}\rangle^2\right)}.\end{aligned} \tag{S5}$$

Note that the left-hand side of Eq. (S5) has only the diagonal elements of correlation matrix. We may concentrate the correlation information onto the diagonal terms by taking a singular value decomposition of the correlation matrix $C_{nn'}^{TLOOs}$ ($n^2\times n'^2$ real matrix) using certain orthogonal matrices O_n^A and $O_{n'}^B$. Under this transformation, TLOOs remain TLOOs as already shown and the left-hand side of Eq. (S5) becomes a trace norm of the new correlation matrix. Using also the invariance of sum of squares of expectation values of TLOOs, the inequality (S5) corresponds to the inequality (10) of main text.

S2. Expectation values of observables in Fock space

In order to calculate the expectation values of Fock-basis observables for a two-mode Gaussian state, the multivariable Hermite polynomials are very useful [5]. The multivariable (4 variables in our case) Hermite polynomials are given by

$$H^{\{R,\theta\}}_{m_1,m_2,n_1,n_2}(y_1,y_2,y_3,y_4) = (-1)^{m_1+m_2+n_1+n_2} \exp\left[\vec{y}^T R \vec{y}\right] \frac{\partial^{m_1}}{\partial y_1^{m_1}} \frac{\partial^{m_2}}{\partial y_2^{m_2}} \frac{\partial^{n_1}}{\partial y_3^{n_1}} \frac{\partial^{n_2}}{\partial y_4^{n_2}} \exp\left[-\vec{y}^T R \vec{y} - \theta \vec{y}\right], \tag{S6}$$

where $\vec{y} = (y_1, y_2, y_3, y_4)^T$. The matrix R is a 4×4 matrix related to the covariance matrix of the two-mode Gaussian state. With these Hermite polynomials, the matrix elements in Fock basis are given by [5]

$$_A\langle m_1|_B\langle m_2|\rho_{AB}|n_1\rangle_A|n_2\rangle_B = \frac{4H^{\{R,0\}}_{m_1,m_2,n_1,n_2}(0,0,0,0)}{\sqrt{m_1!m_2!n_1!n_2!}\sqrt{\det(\gamma_{AB}+\mathbb{1})}}, \tag{S7}$$

where $R = BU\left[(\gamma_{AB}+\mathbb{1})^{-1} - \frac{1}{2}\mathbb{1}\right]U^\dagger D$ and

$$U = \frac{1}{\sqrt{2}}\begin{pmatrix} 1 & i & 0 & 0 \\ 1 & -i & 0 & 0 \\ 0 & 0 & 1 & i \\ 0 & 0 & 1 & -i \end{pmatrix}, \quad B = \begin{pmatrix} 1 & 0 & 0 & 0 \\ 0 & 0 & 1 & 0 \\ 0 & 1 & 0 & 0 \\ 0 & 0 & 0 & 1 \end{pmatrix}, \quad D = \begin{pmatrix} 0 & 0 & 1 & 0 \\ 1 & 0 & 0 & 0 \\ 0 & 0 & 0 & 1 \\ 0 & 1 & 0 & 0 \end{pmatrix}. \tag{S8}$$

Now let us consider a covariance matrix γ_{AB} in a standard form

$$\gamma_{AB} = \begin{pmatrix} a & 0 & c_1 & 0 \\ 0 & a & 0 & -c_2 \\ c_1 & 0 & b & 0 \\ 0 & -c_2 & 0 & b \end{pmatrix}. \tag{S9}$$

In this case the R matrix in equation (S7) is given by

$$R = \frac{1}{2}\begin{pmatrix} \tilde{a}_1 & \tilde{c}_1 & \tilde{a}_2 & \tilde{c}_2 \\ \tilde{c}_1 & \tilde{b}_1 & \tilde{c}_2 & \tilde{b}_2 \\ \tilde{a}_2 & \tilde{c}_2 & \tilde{a}_1 & \tilde{c}_1 \\ \tilde{c}_2 & \tilde{b}_2 & \tilde{c}_1 & \tilde{b}_1 \end{pmatrix}, \tag{S10}$$

where

$$\begin{aligned}
\tilde{a}_1 &= \frac{(b+1)\left(c_1^2-c_2^2\right)}{\left[(a+1)(b+1)-c_1^2\right]\left[(a+1)(b+1)-c_2^2\right]}, \\
\tilde{a}_2 &= -1 + \frac{(b+1)\left[2(a+1)(b+1)-\left(c_1^2+c_2^2\right)\right]}{\left[(a+1)(b+1)-c_1^2\right]\left[(a+1)(b+1)-c_2^2\right]}, \\
\tilde{b}_1 &= \frac{(a+1)\left(c_1^2-c_2^2\right)}{\left[(a+1)(b+1)-c_1^2\right]\left[(a+1)(b+1)-c_2^2\right]}, \\
\tilde{b}_2 &= -1 + \frac{(a+1)\left[2(a+1)(b+1)-\left(c_1^2+c_2^2\right)\right]}{\left[(a+1)(b+1)-c_1^2\right]\left[(a+1)(b+1)-c_2^2\right]}, \\
\tilde{c}_1 &= \frac{-[(a+1)(b+1)-c_1c_2](c_1+c_2)}{\left[(a+1)(b+1)-c_1^2\right]\left[(a+1)(b+1)-c_2^2\right]}, \\
\tilde{c}_2 &= \frac{-[(a+1)(b+1)+c_1c_2](c_1-c_2)}{\left[(a+1)(b+1)-c_1^2\right]\left[(a+1)(b+1)-c_2^2\right]}.
\end{aligned} \tag{S11}$$

For the covariance matrix γ_{AB}^{TMSV} of a two-mode squeezed vacuum state (TMSV) with squeezing parameter r, the matrix R^{TMSV} is given in a simple form

$$R^{TMSV} = \frac{1}{2}\begin{pmatrix} 0 & -\frac{\sinh 2r}{\cosh 2r+1} & 0 & 0 \\ -\frac{\sinh 2r}{\cosh 2r+1} & 0 & 0 & 0 \\ 0 & 0 & 0 & -\frac{\sinh 2r}{\cosh 2r+1} \\ 0 & 0 & -\frac{\sinh 2r}{\cosh 2r+1} & 0 \end{pmatrix} \tag{S12}$$

If this TMSV goes through loss in mode B only, the matrix R_{LB}^{TMSV} is given by

$$R_{LB}^{TMSV}=\frac{1}{2}\begin{pmatrix} 0 & -\frac{\sqrt{\eta}\sinh 2r}{\cosh 2r+1} & -\frac{(1-\eta)(\cosh 2r-1)}{\cosh 2r+1} & 0 \\ -\frac{\sqrt{\eta}\sinh 2r}{\cosh 2r+1} & 0 & 0 & 0 \\ -\frac{(1-\eta)(\cosh 2r-1)}{\cosh 2r+1} & 0 & 0 & -\frac{\sqrt{\eta}\sinh 2r}{\cosh 2r+1} \\ 0 & 0 & -\frac{\sqrt{\eta}\sinh 2r}{\cosh 2r+1} & 0 \end{pmatrix}, \tag{S13}$$

with η transmittance rate. We can similarly obtain the matrix R_{LG}^{TMSV} for the case of amplification.

Therefore, using the matrix elements in equation (S7), we can calculate the expectation values of any observables $A_k^{(n)}\otimes B_l^{(n')}$ to test our criterion in the main text.

S3. Singular value decomposition of correlation matrix

We here show that violation of the steering criterion inequality (10) of the main text is equivalent to the violation of equation (9) in the main text. In view of **Proposition**, let us assume that a given two-mode state ρ_{AB} violates the inequality

$$\|C_{nn'}^{TLOOs}\|_{tr}\le\sqrt{\left(\langle \mathbb{1}_n^A\rangle-\sum_j\langle A_j^{(n)}\rangle^2\right)\left(n'\langle \mathbb{1}_{n'}^B\rangle-\sum_j\langle B_j^{(n')}\rangle^2\right)}. \tag{S14}$$

This violation means that there exists a set of TLOOs $\left\{\tilde{A}_j^{(n)}\right\}$, $\left\{\tilde{B}_j^{(n')}\right\}$, which satisfies an inequality

$$\sum_j\langle \tilde{A}_j^{(n)}\otimes\tilde{B}_j^{(n')}\rangle-\langle\tilde{A}_j^{(n)}\rangle\langle\tilde{B}_j^{(n')}\rangle>\sqrt{\left(\langle \mathbb{1}_n^A\rangle-\sum_j\langle A_j^{(n)}\rangle^2\right)\left(n'\langle \mathbb{1}_{n'}^B\rangle-\sum_j\langle B_j^{(n')}\rangle^2\right)}. \tag{S15}$$

The sets $\left\{\tilde{A}_j^{(n)}\right\}$ and $\left\{\tilde{B}_j^{(n')}\right\}$ can be chosen by employing the eigenvectors of $C_{nn'}^{TLOOs}\left(C_{nn'}^{TLOOs}\right)^T$ and $\left(C_{nn'}^{LOOs}\right)^T C_{nn'}^{LOOs}$ used for the singular value decomposition

$$O_n^A C_{nn'}^{TLOOs}\left(O_{n'}^B\right)^T=C_{nn',\,singular}^{TLOOs}. \tag{S16}$$

Here $C_{nn',\,singular}^{TLOOs}$ is a diagonal matrix with non-negative elements, and O_n^A and $O_{n'}^B$ are truncated orthogonal matrices of which columns are eigenvectors of $C_{nn'}^{TLOOs}\left(C_{nn'}^{TLOOs}\right)^T$ and $\left(C_{nn'}^{TLOOs}\right)^T C_{nn'}^{TLOOs}$, respectively [6].

Now, let us consider steering criterion in equation (9) of **Theorem** and equation (S4) with the observables in equation (S15). We can then derive the following

$$\begin{aligned}\sum_k^N\delta^2\left(\tilde{A}_k^{(n)}\otimes\mathbb{1}+g\mathbb{1}\otimes\tilde{B}_k^{(n')}\right) &=\sum_k\delta^2\left(\tilde{A}_k^{(n)}\right)+g^2\sum_k\delta^2\left(\tilde{B}_k^{(n')}\right)+2g\sum_k\left(\langle\tilde{A}_k\otimes\tilde{B}_k\rangle-\langle\tilde{A}_k\rangle\langle\tilde{B}_k\rangle\right)\\ &=\frac{-\left(\sum_k\langle\tilde{A}_k^{(n)}\otimes\tilde{B}_k^{(n')}\rangle-\langle\tilde{A}_k^{(n)}\rangle\langle\tilde{B}_k^{(n')}\rangle\right)^2}{\sum_k\delta^2\left(\tilde{B}_k^{(n')}\right)}+\sum_k\delta^2\left(\tilde{A}_k^{(n)}\right)\\ &<\frac{\left(n'\langle\mathbb{1}_{(n')}^B\rangle-\sum_k\langle\tilde{B}_k^{(n')}\rangle^2\right)\left(\sum_k\langle A_k^{(n)}\rangle^2-\langle\mathbb{1}_n^A\rangle\right)}{n'\langle\mathbb{1}_{(n')}^B\rangle-\sum_k\langle\tilde{B}_k^{(n)}\rangle^2}+n\langle\mathbb{1}_n^A\rangle-\sum_k\langle A_k^{(n)}\rangle^2\\ &=(n-1)\langle\mathbb{1}_n^A\rangle,\end{aligned} \tag{S17}$$

where we used optimal $g=-\frac{\sum_k\langle\tilde{A}_k^{(n)}\otimes\tilde{B}_k^{(n')}\rangle-\langle\tilde{A}_k^{(n)}\rangle\langle\tilde{B}_k^{(n')}\rangle}{\sum_k\delta^2\left(\tilde{B}_k^{(n')}\right)}$ as in equation (S4) and $\sum_k\delta^2\left(\tilde{A}_k^{(n)}\right)=n\langle\mathbb{1}_n^A\rangle-\sum_k\langle\tilde{A}_k^{(n)}\rangle^2$, $\sum_k\delta^2\left(\tilde{B}_k^{(n')}\right)=n'\langle\mathbb{1}_{n'}^B\rangle-\sum_k\langle\tilde{B}_k^{(n')}\rangle^2$. The inequality in the third line is given by equation (S15)

which we assumed. In summary, the violation of equation (S14) with TLOOs which are constructed by singular value decomposition in equation (S16) is equivalent to the violation of the steering criterion in equation (9) of **Theorem** with the same TLOOs.

[1] E. G. Cavalcanti, S. J. Jones, H. M. Wiseman, and M. D. Reid, Experimental criteria for steering and the Einstein-Podolsky-Rosen paradox, Phys. Rev. A **80**, 032112 (2009).

[2] M. D. Reid, Demonstration of the Einstein-Podolsky-Rosen paradox using nondegenerate parametric amplification, Phys. Rev. A **40**, 913 (1989).

[3] M. Reid, P. Drummond, E. G. Cavalcanti, W. Bowen, P. Lam, H. Bachor, U. Andersen, and G. Leuchs, The Einstein-Podolsky-Rosen paradox: From concepts to applications, Rev. Mod. Phys. **81**, 1727 (2009).

[4] Se-Wan Ji, Jahak Lee, Jiyong Park, and Hyunchul Nha, Steering criteria via covariance matrices of local observables in arbitrary-dimensional quantum systems, Phys. Rev. A **92**, 062130 (2015).

[5] R. Tatham and N. Korolkova, Phys. Rev. A **89**, 012308 (2014).

[6] R. A. Horn and C. R. Johnson, *Matrix Analysis*, Cambridge University Press (*Second Edition*), 2013.
